# Letter from A. J. Miller, M. D.

**Published:** 1868-08-15

**Authors:** A. J. Miller

**Affiliations:** Paris, Ill.


### *CORRESPONDENCE.*

Paris, Ill., *March 23rd*, 1868.

*To the Editor of the Chicago Medical Journal:*

In looking over your Journal of the 1st of March, 1868, my attention was arrested by the novelty of a short article on page 176, from the pen of W. Anderson, M.D., of Leroy, Ill. The article is short, and as I desire to notice it somewhat in detail, I shall beg leave to introduce it entire before making my comments. Its caption is, *Report of a Case of Instrumental Delivery.*

"January 23rd, 1868, I was called, in consultation with Dr. D., to attend a lady, aged 36, who had given birth to seven children. Dr. D. informed me that when he was called in, labor had progressed for twelve hours, the membranes being ruptured and the amniotic fluid having escaped. It was a shoulder presentation, with the head in the left acetabulum, the left arm protruding from the vagina and very much swollen, so that the insertion of the hand into the uterus was impossible, while the force of the uterus was so great as to prevent the adjustment of the fœtus by bringing the head into the pelvis. The arm could not be replaced, and we decided amputation unavoidable. After performing which at the shoulder, the feet were brought down with some difficulty, thus saving any further mutilation of the infant, which, however, we knew to be dead before the operation. The knowledge of its death had no influence on our action, and had it been alive, it would have been born alive, in all probability, with one arm. Can some sage practitioner suggest a method of procedure by which such a danger may be avoided?"

Now, Mr. Editor, while I do not claim to be a sage practitioner, I do claim, in behalf of the profession I am trying to represent, that such cases of so-called instrumental delivery ought to be few and far between. Dr. Anderson fails to inform us as to how long the lady had been in labor, or as to how long the waters had been evacuated at the time of the operation. He says Dr. D. told him she had been in labor twelve hours when he was called in, and that the membranes were ruptured. But as to what time elapsed between the evacuation of the waters and the arrival of Dr. D., and from that time to the time of the performance of the notable operation, we are left wholly in the dark. Nor does he give us any clue to the physical condition of the patient, further than to state that the force of the uterus was so great as to prevent an adjustment of the fœtus by bringing the head into the pelvis. We may then, I think, reasonably infer that several hours had elapsed between the evacuation of the waters and the arrival of Dr. Anderson; in which case we are quite sure no intelligent physician of any practical experience whatever would have ever thought of correcting the presentation by bringing down the child's head? What then? Would he have gone to work to replace the arm? I trow not, and why? Because at that time it was impracticable, and, if practicable, wholly unnecessary. What then ? Would he have decided as did Dr. Anderson and Dr. D., that because the uterine contractions were so forcible as to preclude the possibility of the immediate introduction of his hand into the womb. That, therefore, the amputation of the child's arm was unavoidable. With all due respect for Dr. Anderson and Dr. D., we answer No. What then ?

This question brings us to the solution of the sage problem propounded by Dr. Anderson—the method of procedure. We think, in the first place, that the intelligent and experienced physician would have taken measures to stop the violent contractions of the uterus referred to by the Doctor. First by the exhibition of a full dose of *Opium* or *Laudanum*, and if it need be, *Antimony ;* or if the patient were strong and vigorous, venesection, followed by the *Opium* or *Laudanum*, and nauseating doses of *Antimony ;* but, on the contrary, if the patient were feeble and exhausted, the *Opium* alone would have sufficed. After quieting the pains and allowing the patient to rest for some time, he would have put her (or, at least should), under the full influence of *Chloroform*. The preparatory treatment being thus completed, he would have next proceeded—not to amputate the childs arm, Oh no! certainly not—but to have carefully pushed its shoulder up, and slowly and cautiously introduced his hand by the side of the child's arm, using it for a guide into the uterus, and after finding one or both feet, to have brought them slowly down in accordance with the rules so plainly laid down in our standard text-books ; after which the labor is to be completed as in any other footling case.

Of course the practitioner is to use proper discretion in the use of the remedies referred to, taking into consideration the peculiar conditions, surroundings, and, as he can ascertain, the idiosyncracies of each individual case, and govern himself accordingly. But it may be asked by Dr. Anderson and perhaps others, why use *Chloroform* after the pains have been stopped, and the patient's system relaxed by other remedies.

To this I would answer—first, for the same reason precisely that the surgeon would use it when he is going to perform what would otherwise be a painful operation. To prevent pain and to avoid the shock that would naturally follow so serious an operation, as turning would be under the circumstances above alluded to. And, secondly, because I have used it with great comfort to my patients and perfect satisfaction to myself in cases similar to the one in question.

I remember having been called upon to consult with a Dr. S., of Linton, Indiana, on the 10th of April, 1861. The case was as follows:

Mrs. Sperry; aged 30; the mother of five children; of medium size and somewhat robust constitution. On arriving, I was informed by Dr. S. that he had been in attendance on her for ten hours, and that the waters had been broken for six hours. It was a shoulder presentation—the head resting in the right iliac fossa, and not in the *acetabulum*, as in the case reported by Dr. Anderson. The left arm extruding through the vagina, the back of the child of course looking towards the abdomen of the mother. Upon making an examination I found, as did Dr. Anderson in his case, that the child's arm was verv much swollen, and the uterus perfectly moulded around its body. The soft parts of the mother were dry and hot, and the uterine contractions so forcible as to utterly preclude the idea of the introduction of the hand into the uterus. Upon retiring to consult with the doctor, he informed me that child's arm had made its appearance in the vagina sometime before the breaking of the waters, and to use his own language, "he had put the d—d thing up a dozen times, and at the very next pain it came down again," a result so natural that I at once gave perfect credence to his statements on this point. After occupying himself with these manipulations for some time, the pains became so forcible as to render it impossible for him to *assist* (?) labor in this *way* any longer.

And what next, Mr. Editor, do you suppose he did? Amputate the child's arm? He did not do that either. He gave the poor unfortunate woman a pint of *Ergot Tea* (which, luckily for her, I think was worthless). And what next? Why, he made gentle traction by the child's arm, as he said, to assist the pains in the delivery. Great God! what a fearful responsibility. After pulling and tugging at the child's arm and drenching the mother with *Ergot Tea* for full five hours, of course it (the child) was dead. The immaculate infant fell a victim to his unpardonable ignorance. I say unpardonable ignorance, for I do not think we ought to use soft terms in speaking of such reprehensible practice—a practice which, had the *Ergot* been of good quality, might have resulted in the rupture of the uterus, and consequently the death of the mother.

Now, Mr. Editor, I am happy to state that Dr. S. is not a graduate of Rush Medical College, but of Brush College. Its precise location I am unable to give. But that Dr. S. is a fair specimen of its alumni, there can be no doubt.

But to return to our case. After patiently hearing the Doctor's statements relative to his treatment of the patient, I suggested that we had better put her under the full influence of *Opium*, with the addition of a little *Antimony*, and let her rest for two or three hours. But at his wits' end, he readily assented, and the suggestion was acted on at once.

In a short time she was free from pain, and at the end of time above specified, I put her under the full influence of *Chloroform*. This being done, Dr. S., as previously agreed upon, proceeded to introduce his great rough hand (which had been amply developed in the college aforesaid), into the womb of the patient, while I kept her under the influence of *Chloroform*, and after some twenty or thirty minutes of fruitless research for the child's feet he gave up in despair, and requested me to exchange places with him, which I did. Upon introducing my hand, I succeeded in finding one of the child's knees, and, hooking my index finger into its flexure, I I slowly and cautiously drew it down according to the rules laid down in our text books, yet not without some considerable trouble, for the operation of turning under the circumstances is a perplexing, and sometimes a difficult one. This being done, the delivery of course was easily effected, and within two weeks the mother was able to leave her bed.

We have three objects in view, Mr. Editor, in reporting this case. First, that your readers may see the analogy between it and the one reported by Dr. Anderson. Secondly, that they may become acquainted with the different modes by which uninformed medical men punish poor, confiding unoffending women, and by which they literally slaughter helpless and innocent children. And thirdly, to impress upon their minds the necessity of properly informing themselves that they may thereby avoid such reprehensible practice.

I am aware, Mr. Editor, that this is plain dealing, perhaps rather harsh. But, Sir, ought we not to cry aloud and spare not, while such practice is rife in our land? Are we, I say, for fear of wounding the feelings of our erring brethren, to let such practice go unrebuked, and especially so when the rules for avoiding it are so few, so plain, and so easy of access by every medical man? It is possible that a necessity for evisceration may occur in these cases; but that it is necessary under any circumstances, to amputate the child's arm, we think not.

A. J. Miller, M.D.

